# New Tetromycin Derivatives with Anti-Trypanosomal and Protease Inhibitory Activities †

**DOI:** 10.3390/md9101682

**Published:** 2011-09-26

**Authors:** Sheila M. Pimentel-Elardo, Verena Buback, Tobias A.M. Gulder, Tim S. Bugni, Jason Reppart, Gerhard Bringmann, Chris M. Ireland, Tanja Schirmeister, Ute Hentschel

**Affiliations:** 1 Julius-von-Sachs Institute for Biological Sciences, University of Würzburg, Julius-von-Sachs-Platz 3, Würzburg 97082, Germany; E-Mail: ute.hentschel@uni-wuerzburg.de; 2 Institute for Pharmacy and Food Chemistry, Am Hubland, Würzburg 97074, Germany; E-Mails: verena.buback@uni-wuerzburg.de (V.B.); schirmei@pharmazie.uni-wuerzburg.de (T.S.); 3 Institute of Organic Chemistry, University of Würzburg, Am Hubland, Würzburg 97074, Germany; E-Mails: tgulder@uni-bonn.de (T.A.M.G.); bringman@chemie.uni-wuerzburg.de (G.B.); 4 Department of Medicinal Chemistry, University of Utah, Salt Lake City, UT 84112, USA; E-Mails: tbugni@pharmacy.wisc.edu (T.S.B.); jreppart@gmail.com (J.R.); cireland@pharm.utah.edu (C.M.I.)

**Keywords:** tetromycin, anti-trypanosomal, protease inhibition, Streptomyces axinellae, marine sponge

## Abstract

Four new tetromycin derivatives, tetromycins **1**–**4** and a previously known one, tetromycin B (**5**) were isolated from *Streptomyces axinellae* Pol001^T^ cultivated from the Mediterranean sponge *Axinella polypoides*. Structures were assigned using extensive 1D and 2D NMR spectroscopy as well as HRESIMS analysis. The compounds were tested for antiparasitic activities against *Leishmania major* and *Trypanosoma brucei*, and for protease inhibition against several cysteine proteases such as falcipain, rhodesain, cathepsin L, cathepsin B, and viral proteases SARS-CoV M^pro^, and PL^pro^. The compounds showed antiparasitic activities against *T. brucei* and time-dependent inhibition of cathepsin L-like proteases with *K*_i_ values in the low micromolar range.

## 1. Introduction

Protozoan diseases such as malaria, leishmaniasis, and African and South American trypanosomiasis afflict millions of people particularly in many tropical and subtropical countries. Coronaviruses are also important pathogens that mainly cause respiratory and enteric diseases in humans. For example, SARS-CoV causes severe acute respiratory syndrome (SARS) resulting in the epidemic in 2002–2003 with more than 8000 death cases worldwide [1]. The increasing resistance to current therapies and the toxicities of existing drugs aggravates the prevalence of these infectious diseases. These issues thus necessitate the need for alternative therapies. A promising strategy is to develop new drugs targeting the parasites’ cysteine proteases that are crucial for their growth, differentiation and pathogenicity. Examples of these proteolytic enzymes are rhodesain from *Trypanosoma rhodesiense*, falcipain from *Plasmodium falciparum*, and cysteine cathepsins from *Leishmania* spp. [2]. Moreover, the coronavirus main protease (M^pro^, also named 3C-like protease 3CL^pro^) as well as the papain-like protease (SARS-CoV PL^pro^) are also considered to be major targets for new antiviral drugs against SARS and other coronavirus infections [3].

Marine sponges are the most prolific sources of bioactive metabolites in the marine environment. More than 5300 different natural products have so far been isolated from marine sponges and their associated bacteria and more than 200 new metabolites are reported from sponges annually [4]. Owing to their filter-feeding capacity and to the frequent presence of massive amounts of microbial symbiotic consortia in the mesohyl tissues, sponges are also rich sources of microorganisms, many of which produce interesting bioactivities [5,6]. Cultivation efforts have focused particularly on the actinomycetes, as this taxonomic clade is responsible for the production of about half of the discovered antibiotics to date [7–9]. We have recently described the isolation of a new actinomycete species, *Streptomyces axinellae* type strain Pol001 cultivated from the Mediterranean sponge, *Axinella polypoides* [10]. Here, the isolation of new tetromycin derivatives with unprecedented inhibitory activities against several clinically important cysteine proteases is reported. The choice to use cysteine proteases as possible target enzymes is based on the tetronic acid moiety which is part of all the isolated metabolites. Due to its lactone ring with the double bond activated for nucleophilic attack by its electron withdrawing substituents, this moiety is predestined to inhibit cysteine proteases.

## 2. Results and Discussion

### 2.1. Structure Elucidation

*Streptomyces axinellae* Pol001^T^ was grown on MS [11] agar and the secondary metabolites were extracted with ethyl acetate. HPLC fractionation yielded five compounds, tetromycins 1–4 (**1**–**4**), and tetromycin B (**5**) [12]. HRESIMS and extensive 1D and 2D NMR analysis (Table 1) suggested that the compounds were new constitutional isomers with several tetromycins that had been previously isolated from a *Streptomyces* sp. strain MK67-CF9 [13]. Tetromycin **1** displayed a pseudo-molecular ion in the positive ESIMS spectrum at *m/z* 910.4388 [M + Na]^+^ that corresponded to a molecular formula of C_50_H_65_NO_13_ and was isomeric with tetromycin C5 [13]. Tetromycin **2** was observed to have a molecular ion in the positive ESIMS spectrum at *m/z* 911.4232 [M + Na]^+^ corresponding to a molecular formula of C_50_H_64_O_14_ which was isomeric with tetromycin C1 [13] and tetromycin **4** with *m/z* 897.4066 [M + Na]^+^ matching a molecular formula of C_49_H_62_O_14_ which was isomeric with tetromycin C2 [13].

**Figure 1:**
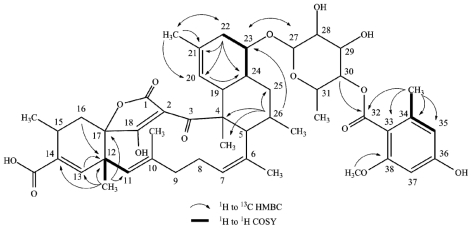
Selected 2D NMR correlations in tetromycin **4**.

The IR spectra of tetromycins **1**–**4** showed the expected absorption bands for hydroxy groups (~3200 cm^−1^), acid functions (~2800 cm^−1^), carbonyl groups (~1700 cm^−1^), conjugated carbonyl groups (~1640 cm^−1^), C–C-double bonds (~1300 cm^−1^), and aromatic rings (~1600 cm^−1^). The presence of these functional groups was further supported by the ^1^H, ^13^C and DEPT NMR data. The carbon-proton connectivities were unambiguously assigned by HSQC experiments. In all four compounds, the ^13^C NMR indicated the presence of a two-times hydroxy/alkoxy-substituted aromatic moiety made of carbons C33–38. The corresponding signals in the ^1^H NMR spectra showed only two aromatic protons (H35; H37) in *meta*-position to each other of the aromatic ring (see Table 1 for shifts, multiplicity, and coupling constants). HMBC correlations of the methyl group protons H34′ to the carbonyl C32 and to C33–C35 of the aromatic ring confirmed an additional *ortho*-substitution pattern. COSY interactions of H34′ to only one of the aromatic protons (H35) left C36 and C38 open for further substitution. Aromatic carbon shifts of C36 and C38 at ~160 ppm indicated the hydroxy or alkoxy substituents to be located at these positions. In tetromycins **2**, **3**, and **4**, the hydroxy group at C38 is methylated, as evident from the correlations between methoxy group protons H38′ and C38 in the HMBC spectrum. By contrast, compound **1** is hydroxylated at C38. C36 carries a methoxy group in compounds **1** and **2** and hydroxy groups in **3** and **4** as clearly indicated by HMBC, ^13^C and ^1^H data (Figure 1).

HMBC correlations of H30 to C32 in tetromycins **1**–**4** revealed the connectivity of the aromatic ring to a pyran fragment via an ester or amide bond, respectively (Figure 1). The substitution pattern of this moiety was unambiguously established by HMBC and ROESY data. The connectivity of the pyran group to the decalin ring system consisting of C4, C5, C19–C26 via the acetal in *para*-position to the ester/amide junction was detected by bilateral HMBC correlations between carbons and protons C/H27 and C/H23 (Figure 1). The so far described fragments are in agreement with the published structures of tetromycins [13].

**Figure 2:**
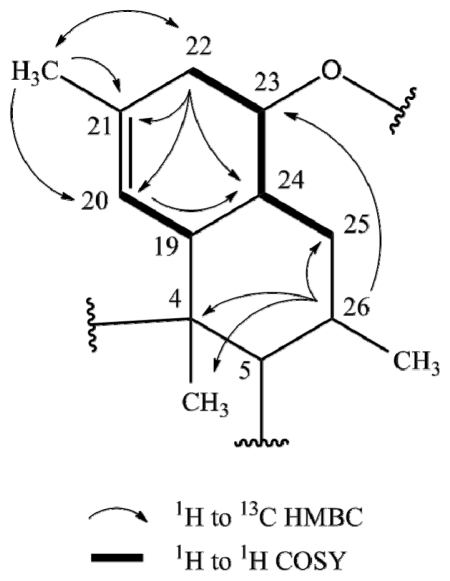
Selected 2D NMR correlations in decalin of tetromycin **4**.

The main differences between tetromycins **1**, **2**, and **4** and their previously isolated congeners covered in a patent [13] lie in the presence of a double bond between C20 and C21 (instead of between C25 and C26 as stated for tetromycin C5) of the decalin ring system and the position of a methyl group at C21 instead of at C20. The following detailed NMR correlations accounting for these altered positions are exemplarily discussed for tetromycin **4** using selected key NMR interactions (Figure 2). HSQC spectra showed two diastereotopic protons H22 (δ 2.26, 1.95) attached to C22 (δ 39.7). Strong ROESY and COSY correlation of H22 (δ 2.26) to H23 (δ 3.39) and correlative coupling constants suggested a ^3^*J*-coupling and a direct connection between C23 and C22. In the HMBC H22 (δ 1.95) correlates with a methyl carbon at δ 18.9 (C21′) and two vinylic carbons at δ 125.8 (C20) and 140.4 (C21), the latter presumably carrying the methyl group in agreement with the higher shift and according to HMBC signals. Methyl protons H21′ (δ 1.69) showed three strong correlations, not only with vinylic carbons C21 and C20 but also with C22 suggesting again the positioning of the methyl group at C21 and not at C20 as described earlier [13]. This conclusion was further substantiated by a strong HMBC correlation of the vinylic proton H20 (δ 4.92) with C24 (δ 46.5). Closing the first six-membered ring of the decalin system, C24 is directly connected to C23 as evidenced by direct COSY interactions and HMBC correlations of H22 (δ 2.26) to C24. C24 also correlated with proton H26 (δ 2.09), which in turn showed HMBC correlation with C23 (δ 84.9), C25 (δ 37.8), C4 (δ 52.6) and C4′ (δ 15.6). Looking at the corresponding ^13^C NMR shifts, no C-C double bond could be located in the second six-membered ring of the decalin system, specifically not between C25 and C26 as featured in the previously described compounds. HMBC, ^13^C, and ^1^H data confirmed this result. We have thus discovered a series of tetromycin derivatives possessing a double bond between C20 and C21 and a methyl group at C21. The remaining partial structures of compounds **1**, **2**, and **4** are in turn in accordance with the already published compounds.

**Figure 3:**
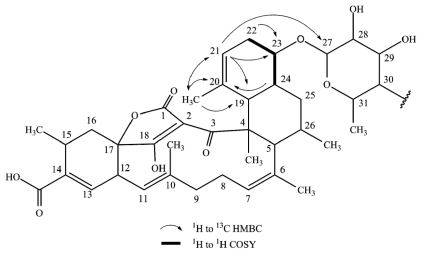
Selected 2D NMR correlations in decalin of tetromycin **3**.

Interestingly, tetromycin **3** exhibiting a molecular ion in the positive ESIMS spectrum at *m/z* 861.4062 [M + H]^+^ corresponding to a molecular formula of C_48_H_61_O_14_ was found to be a completely new derivative. The differences are again found in the decalin ring system made of C4, C5, C19–C26 and additionally in the cyclohexene fragment (C12–C17) carrying the acid group at C14 (Figure 3). As seen for tetromycins **1**, **2**, and **4**, HSQC data of tetromycin **3** revealed two diastereotopic protons H22 (δ 1.93, 2.32) located at C22 (δ 34.1). HMBC correlation of C23 with H22 (δ 1.93) again accounted for the single bond between C23 and C22 as discussed before. However, in this compound C23 also shows strong HMBC correlation to vinylic proton H21 (δ 4.95), suggesting close proximity to the carbon at δ 120.3 (C21) which is a CH-unit according to DEPT data. H21 furthermore correlates with methyl group C20′ (δ 14.3), C19 (δ 42.1), and interestingly also with C27 (δ 103.1), which reinforces the proposition of assigning δ 120.3 to C21 instead of to C20 as was found in our other isolated compounds. Unobserved in tetromycins **1**, **2** and **4**, methyl group protons H20′ of **3** correlated with C20, C21, and also with C19 (δ 42.07), the bridge atom of both six-membered rings of the decalin system, leading to the undoubted conclusion about the methyl group position at C20. The second six-membered ring only features saturated carbon atoms as was discussed above.

**Figure 4:**
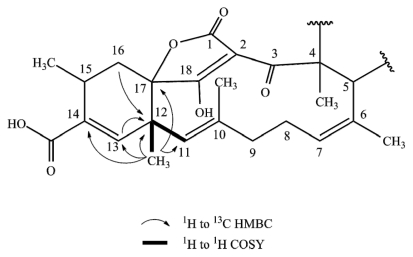
Selected 2D NMR correlations in the cyclohexene fragment of tetromycin **4**.

All tetromycin derivatives possess a cyclohexene fragment made of C12–17, with C12 and C17 being the bridge atoms to the adjacent ring systems and C14 carrying the acid functional group. There is a C–C-double bond present between C13 and C14 as was already described in the published patent. Bridge atom C12 in the published compounds and in our compounds **1**, **2**, **4**, and **5** is a quaternary carbon carrying methyl group C12′ (see Table 1 for respective shifts) as unmistakably revealed by COSY coupling of H11 and H12′, HMBC correlation of C12′ with H13 and H16 and those of H12′ with C13, C14, C17, C11, and C12 (Figure 4).

**Figure 5:**
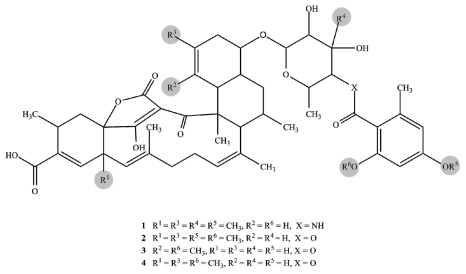

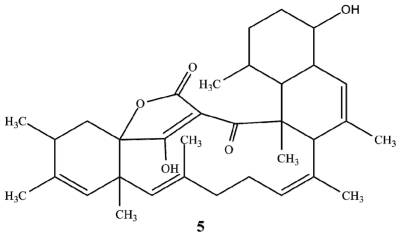
Structures of tetromycins **1**–**4** and tetromycin B (**5**).

After unambiguously confirming all positions of the cyclohexene carbons of tetromycin **3**, none of the named correlations to a methyl group could be found. Furthermore, C12 showed a lower shift than in the other compounds and appeared as a CH in the DEPT spectrum. The loss of the methyl group at C12 was in accordance with the observed lower molecular mass, also indicating a formal loss of a CH_2_-fragment compared to the other compounds. We therefore report the discovery of the new tetromycin derivative (**3**) featuring a double bond between C20 and C21, a methyl substituent at C20, and hydrogen at C12. Lastly, compound **5** was found to be identical with tetromycin B [6] with *m/z* 557.3229 [M + Na]^+^ corresponding to a molecular formula of C_34_H_46_O_5_. Figure 5 shows the structures of tetromycins **1**–**4** and tetromycin B (**5**).

### 2.2. Antiparasitic Activities

The compounds were tested against the parasites *Leishmania major* and *Trypanosoma brucei* subsp. *brucei* and for cytotoxicity against 293T kidney epithelial cells and J774.1 macrophages (Table 2). Anti-*Leishmania* activity was only found for tetromycin **3**. All five compounds exhibited anti-*Trypanosoma* activities at both 48 and 72 h time points as judged by IC_50_ values below 100 μM. The compounds also showed cytotoxic activities with IC_50_ < 100 μM against the kidney cell line and macrophages with the notable exception of tetromycin **1**.

### 2.3. Protease Inhibition

**Figure 6:**
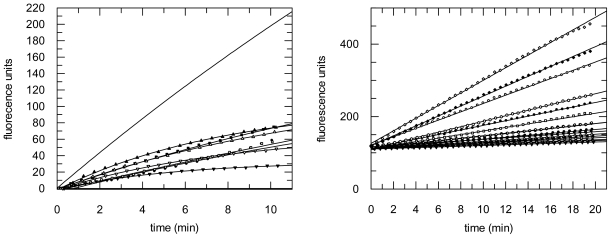
Inhibition of rhodesain (left, time-dependent inhibition) and cathepsin B (right, non-time dependent inhibition) by tetromycin B (**5**) (inhibitor concentrations from top to bottom: 0, 1.67, 3.34, 6.68, 8.35, 10.0, 13.4, 16.7 μM).

The compounds were further subjected to protease inhibition assays with mammalian proteases cathepsin B and L, parasite cathepsin-L like proteases falcipain-2 (from *P. falciparum*) and rhodesain (from *T. brucei rhodesiense*), and with the coronaviral papain-like protease SARS-CoV PL^pro^ as well as with the coronaviral main protease SARS-CoV M^pro^. Initial screening assays were performed at 100 μM compound concentration. In a second step, compounds showing >50% enzyme inhibition at this concentration (compounds **3**–**5**) were analyzed in detail. Substrate hydrolysis was monitored over 10–40 min in the absence or presence of various inhibitor concentrations (seven inhibitor concentrations spanning from zero to total inhibition of the respective enzyme). Interestingly, inhibition of the cathepsin L-like proteases was observed to be time-dependent, while inhibition of cathepsin B and the coronaviral papain-like protease was not (Figure 6). All compounds were inactive or only weakly active (<10% inhibition at 100 μM) against the SARS-CoV M^pro^.

**Figure 7:**
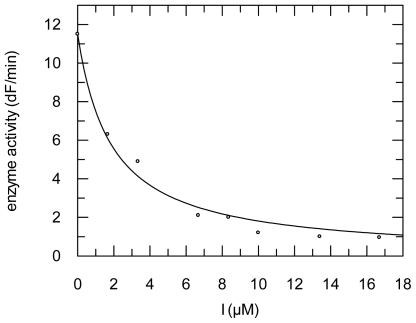
Inhibition of cathepsin B by tetromycin B (**5**). A *K*_i_ value of 1.50 μM was obtained.

For cathepsin B and SARS-CoV PL^pro^, dissociation constants of enzyme-inhibitor complexes were calculated using the Dixon equation by fitting the residual enzyme activities against the inhibitor concentrations (Figure 7) and by correction to zero-substrate concentration (see Experimental Section).

**Figure 8:**
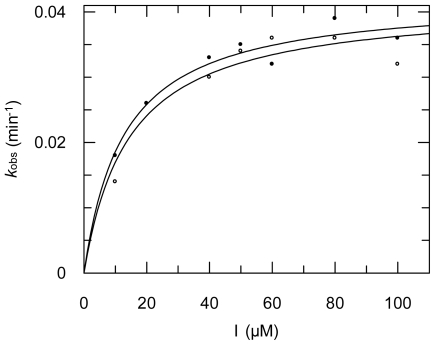
Inhibition of rhodesain by compound **3**; data from two independent assays are shown. The following inhibition constants were obtained: *K*_i_ = 2.1 μM; *k*_i_ = 0.042 min^−1^; *k*_2nd_ = 38,100 M^−1^ min^−1^.

In cases of time-dependent inhibition the pseudo-first order rate constants of inhibition *k*_obs_ (obtained from the progress curves) were fitted against the inhibitor concentrations to obtain *K*_i_, *k*_i_, and finally *k*_2nd_ values (Figure 8, see Experimental Section for details).

In order to ascertain if inhibition was competitive with respect to the substrates, we determined the *K*_i_ values for inhibition of rhodesain by tetromycin B (**5**) at three different substrate concentrations, and found no significant differences, indicating competitive inhibition.

Interestingly, time-dependent inhibition was found only with cathepsin-L like enzymes, not with cathepsin B or the coronaviral protease PL^pro^. This observed time-dependent inhibition could either be due to an irreversible inhibition mechanism resulting from covalent reaction of the reactive moiety of the inhibitors, the tetronic acid containing an α,β-unsaturated lactone ring, with the cysteine residue of the active sites of the target proteases, or to a slow-binding mechanism resulting from conformational changes of the enzyme-inhibitor complex. The reasons for the differences between cathepsin-L like proteases (mammalian cathepsin L, falcipain, rhodesain) and the others are not yet clear and will require more detailed studies with smaller synthetic derivatives containing the tetronic acid moiety.

The inactivity against the SARS-CoV M^pro^ which is a cysteine protease differing from the other target proteases by the protonation state of its active site (Cys-SH/His instead of ion pair Cys-S^−^/His-H^+^) and its three-dimensional fold (similar to the serine protease chymotrypsin instead of papain-like fold) shows that the compounds are not unselective frequent hitters. It also supports the hypothesis that the compounds are covalent inhibitors of cathepsin-L like proteases, since the active centre of these proteases is more nucleophilic and thus better capable for reaction with the tetronic acid moiety. The inhibition constants (*k*_2nd_ values and *K*_i_ values) do not differ largely between the three compounds indicating a common inhibition mechanism. Table 3 summarizes the inhibition constants.

## 3. Experimental Section

### 3.1. General Experimental Procedures

High resolution ESIMS analyses were performed on a Micromass Q-Tof micro mass spectrometer. NMR spectra were obtained on Varian INOVA 500 (^1^H: 500 MHz, ^13^C: 125 MHz) and Varian INOVA 600 (^1^H: 600 MHz, ^13^C: 150 MHz) spectrometers with a 3 mm Nalorac MDBG probe and a 5 mm cold probe, respectively. UV spectra were acquired in spectroscopy grade MeOH using a Hewlett-Packard 8452A diode array spectrophotometer. IR spectra were recorded using a JASCO FT/IR-400 spectrophotometer. HPLC was performed on an Agilent 1100 system using a Luna C_18_ (Phenomenex, Inc.) (250 × 10 mm, 5 μm) column.

### 3.2. Biological Material

*Streptomyces axinellae* strain Pol001^T^ was cultivated from the Mediterranean sponge *Axinella polypoides* [10]. The sponge was collected by scuba diving offshore from Banyuls-sur-Mer, France (GPS: 42°29′ N 03°08′E). *Streptomyces axinellae* strain Pol001^T^ is deposited at the Deutsche Sammlung von Mikroorganismen und Zellkulturen GmbH (DSMZ 41948T) and the Collection de l’Institut Pasteur (CIP 109838T).

### 3.3. Extraction and Isolation

The bacterial strain was grown on 200 MS [11] agar plates at 30 °C for seven days. Mycelial mass together with the agar were cut into small pieces and macerated overnight with sufficient volume of ethyl acetate to fully submerge the biomass. The resulting solution was filtered and maceration with ethyl acetate was repeated. Both filtrates were combined and dried by rotary evaporation. The isolation of the compounds was carried out by semi-preparative HPLC using H_2_O + 0.1% TFA (A) and CH_3_CN (B) as the solvents and the following gradient: flow 4.5 mL/min; 0–10 min 90% B, 11–15 min 100% B to yield five compounds: tetromycin 1 (**1**, 2.7 mg, *Rt* = 5.94 min); tetromycin 2 (**2**, 4.4 mg, *Rt* = 7.75 min); tetromycin 3 (**3**, 2.2 mg, *Rt* = 9.67 min); tetromycin 4 (**4**, 4.4 mg, *Rt* = 12.24 min); tetromycin B (**5**, 2.1 mg, *Rt* = 17.47 min).

Tetromycin 1 (**1**): light yellow amorphous solid; [α]^20^ _D_ −40.2 (*c* 1.92, MeOH); UV (MeOH) λ_max_ (log ɛ) 210 (1.41), 244 (0.77), 268 (0.54); ^1^H and ^13^C NMR data, see Table 1; ESIMS *m/z* 910.4388 [M + Na]^+^ (calcd for C_50_H_65_NO_13_Na, 910.4354).

Tetromycin 2 (**2**): light yellow amorphous solid; [α]^20^ _D_ −38.8 (*c* 2.50, MeOH); UV (MeOH) λ_max_ (log ɛ) 212 (1.49), 242 (0.70), 268 (0.54); ^1^H and ^13^C NMR data, see Table 1; ESIMS *m/z* 911.4232 [M + Na)]^+^ (calcd for C_50_H_64_O_14_Na, 911.4219).

Tetromycin 3 (**3**): light yellow amorphous solid; [α]^20^ _D_ −47.7 (*c* 3.00, MeOH); UV (MeOH) λ_max_ (log ɛ) 214 (1.60), 244 (0.72), 266 (0.85); ^1^H and ^13^C NMR data, see Table 1; ESIMS *m/z* 861.4062 [M + H]^+^ (calcd for C_48_H_61_O_14_, 861.4062).

Tetromycin 4 (**4**): light yellow amorphous solid; [α]^20^ _D_ −44.1 (*c* 3.83, MeOH); UV (MeOH) λ_max_ (log ɛ) 214 (1.84), 246 (0.79), 266 (1.03); ^1^H and ^13^C data, see Table 1; ESIMS *m/z* 897.4066 [M + Na]^+^ (calcd for C_49_H_62_O_14_Na, 897.4037).

Tetromycin B (**5**): light yellow amorphous solid; [α]^20^ _D_ −12.0 (*c* 1.83, MeOH); UV (MeOH) λ_max_ (log ɛ) 210 (1.42), 244 (0.59), 268 (0.55); ESIMS *m/z* 557.3229 [M + Na]^+^ (calcd for C_34_H_46_O_5_Na, 557.3243).

### 3.4. Antiparasitic Activity Assays

*Leishmania major* promastigotes were seeded at a cell density of 1 × 10^7^ cells/mL into 96-well plates in complete medium (RPMI with NaHCO_3_, 10% FCS, 2 mM glutamine, 10 mM Hepes pH 7.2, 100 U/mL penicillin, 50 μg/mL gentamicin, 50 mM 2-mercaptoethanol) without phenol red (200 mL), in the absence or presence of different concentrations of the compounds. These were then incubated for 24 h at 26 °C, 5% CO_2_ and 95% humidity. Following the addition of 20 μL of Alamar Blue, the plates were incubated again and the optical densities (ODs) measured 24 and 48 h later with an enzyme-linked immunosorbent assay (ELISA) reader (Multiskan Ascent, Germany) using a test wavelength of 540 nm and a reference wavelength of 630 nm. Absorbance in the absence of compounds was set as 100% of growth. Amphotericin B was used as a reference compound and positive control. The effects of cell density, incubation time and the concentration of DMSO were examined in control experiments. The final concentration of DMSO in the medium never exceeded 1% vol/vol and had no effect on the proliferation of extracellular or intracellular parasites. For every experiment, each drug concentration was assayed in duplicate wells [14].

Trypomastigote forms of *Trypanosoma brucei* subsp. *brucei* laboratory strain TC 221 were cultured in complete Baltz medium [80 mL Baltz medium basic solution, 0.8 mL 2-mercaptoethanol stock solution (20 mM), 0.8 mL penicillin/streptomycin (10,000 U/mL), 16 mL FCS (inactivated for 30 min at 56 °C)]. Baltz medium basic solution is composed of the following: 500 mL MEM with Earle’s salts and l-glutamine, 3 g Hepes, 0.5 g monohydrate glucose, 0.110 g sodium pyruvate, 0.007 g hypoxanthine, 0.002 g thymidine, 0.0107 g adenosine, 0.0141 g bathocuproinedisulfonic acid disodium salt, 0.146 g glutamine, 5 mL sterile non-essential amino acid concentrate (100×, pH 7.5). A defined number of parasites (10^4^ trypanosomes per mL) in test chambers of 96-well plates were exposed to various concentrations of the test substances (dissolved in DMSO) to make a final volume of 200 μL in duplicate. Positive (trypanosomes in culture medium) and negative controls (test substance without trypanosomes) were run simultaneously with each plate. The plates were then incubated at 37 °C in an atmosphere of 5% CO_2_ for a total time period of 72 h. After 24 h, 20 μL of Alamar Blue were added. The activity of the test substances was measured by light absorption using MR 700 Microplate Reader at a wavelength of 550 nm with a reference wavelength of 630 nm. The first reading was done at 48 h and subsequently at 72 h. The effect of the test substances was quantified in IC_50_ values by linear interpolation of three independent measurements [15].

### 3.5. Cytotoxicity Assays

J774.1 macrophages were cultured in complete medium (RPMI with NaHCO_3_, 10% FCS, 2 mM glutamine, 10 mM Hepes pH 7.2, 100 U/mL penicillin, 50 μg/mL gentamicin, 50 M 2-mercaptoethanol) without phenol red in the absence or presence of increasing concentrations of the compounds at a cell density of 1 × 10^5^ cells/mL (200 μL) for 24 h at 37 °C, 5% CO_2_ and 95% humidity. Following the addition of 20 μL of Alamar Blue, the plates were incubated and the ODs measured at 24, 48 and 72 h. The same Alamar Blue assay previously described for *Leishmania* was followed. Kidney epithelial 293T cells were tested in the same manner as the macrophages but using complete DMEM medium (4.5 g/L solution of DMEM high glucose solution with sodium pyruvate but without l-glutamine, FBS superior at final concentration of 20%, 200 mM l-glutamine 100×) and cell density (2 × 10^4^ cells/mL).

### 3.6. Protease Inhibition Assays

The fluorometric enzyme assays were performed on a Cary Eclipse fluorescence spectrophotometer (Varian, Darmstadt, Germany) using a microplate reader (excitation 365 nm, emission 460 nm). Cathepsin B and L protease inhibition assays were performed at 25 °C in 50 mM Tris-HCl buffer pH 6.2, containing 5 mM EDTA, 2 mM DTT, 200 mM NaCl, 0.005% Brij 35 as described by Vicik *et al.* [16–19].

Assays with falcipain-2 and rhodesain were performed in 50 mM acetate buffer pH 5.5 with 5 mM DTT, 5 mM EDTA, 200 mM NaCl, and 0.005% Brij 35. The substrate (Cbz-Phe-Arg-AMC for all four enzymes) and inhibitor stock solutions were prepared in DMSO (10% final concentration) and were diluted with assay buffer. The final substrate concentrations for the inhibition assays were 10.0 μM (RD), 25 μM (FP-2), 6.25 μM (CL), and 100 μM (CB).

The fluorometric SARS-CoV PL protease inhibition assays were performed at 25 °C in a 20 mM Tris-HCl buffer pH 7.5, containing 0.1 mM EDTA, 1 mM DTT, 200 mM NaCl, and 0.005% Brij 35. The final substrate concentration (Z-Arg-Leu-Arg-Gly-Gly-AMC-acetate salt) was 50 μM. Assays with SARS-CoV main protease were done in 20 mM Tris buffer pH 7.5 with 0.1 mM EDTA, 1 mM DTT, and 200 mM NaCl. The substrate used was Abz-Ser-Val-Thr-Leu-Gln-Ser-Tyr(NO_2_)-Arg, TFA salt (Abz, anthranilic acid) at 50 μM final substrate concentration (excitation 325 nm, emission 425 nm).

First, compounds were tested at 100 μM final concentration. For compounds showing considerable inhibition at this concentration (>50%), inhibition kinetics were analyzed in detail. Progress curves of substrate hydrolysis in absence or presence of inhibitor were monitored over a period of 10–40 min. For compounds showing non-time dependent inhibition the residual enzyme activities *vi* (obtained from the slopes of the progress curves) were fit to the inhibitor concentrations using the Dixon equation *vi* = *vo*/(1 + I/*K*_i_ ^app^), and correction to zero substrate concentration by the Cheng-Prusoff equation [20,21] for competitive inhibitors: *K*_i_ = *K*_i_ ^app^/(1 + [*S*]/*K*_m_). For compounds showing time-dependent inhibition the progress curves were fit to the exponential equation: *y* = limit × (1 − exp(−*k*_obs_ × *t*)) + offset yielding the pseudo-first order rates of inhibition *k*_obs_. These were then fit to the inhibitor concentrations with *k*_obs_ = *k*_i_ × *I*/(*K*_i_ ^app^ + *I*) yielding *k*_i_ and *K*_i_ ^app^; *K*_i_ ^app^ values were corrected to zero substrate concentration by: *K*_i_ = *K*_i_ ^app^/(1 + [*S*]/*K*_m_) and *k*_2nd_ was calculated from *k*_2nd_ = (*k*_i_/*K*_i_). The *K*_m_ values used to correct *K*_i_ ^app^ values were determined in previous work: 0.9 μM (RD), 21.5 (FP-2), 6.5 (CL), 150 (CB), 850 (SARS-CoV PL^pro^). The program GraFit (Erithacus Software Ltd., version 5.0.13) was used to calculate the inhibition constants.

## 4. Conclusions

These results presented herein highlight the importance of marine actinomycetes for drug discovery, as shown here by the identification of four structurally new tetromycin derivatives and a previously known compound, tetromycin B, from the marine sponge-derived isolate, *Streptomyces axinellae*. Antiparasitic activities against the causative agent of African sleeping disease, *Trypansoma brucei*, were reported. Furthermore, the compounds showed time-dependent inhibition of cathepsin L-like proteases with *K*_i_ values in the low micromolar range.

The selectivity for cathepsin-like enzymes gives a good starting point for further studies with the aim to elucidate the moiety of the inhibitors responsible for inhibition (probably the tetronic acid moiety), to improve selectivity between mammalian and parasite enzymes, which may lead to less cytotoxicity, and to enhance inhibition potency. Furthermore, additional targets within the parasites, have to be taken into account. In *Trypanosoma brucei*, e.g., the cathepsin-B like protease TbCatB is also known to be essential for the parasite’s life cycle, and activity against this protease could contribute to the antiparasitic activities of the compounds. Finally, future work will also address stereochemical aspects.

## Figures and Tables

**Table 1:** ^13^C and ^1^H NMR data of tetromycins **1**–**4** in acetone-*d*_6_.

Posn.	1				2				3				4			

	δ_C_	mult	δ_H_	mult, (*J*_H-H_)	δ_C_	mult	δ_H_	mult, (*J*_H-H_)	δ_C_	mult	δ_H_	mult, (*J*_H-H_)	δ_C_	mult	δ_H_	mult, (*J*_H-H_)

1	167.4	C			168.4	C			174.8	C			169.0	C		
2	96.8	C			99.6	C			96.8	C			99.1	C		
3	206.3	C			204.5	C			205.4	C			204.6	C		
4	52.4	C			52.7	C			52.6	C			52.6	C		
4′	15.8	CH_3_	1.52	s	16.0	CH_3_	1.52	s	15.4	CH_3_	1.54	s	15.6	CH_3_	1.52	s
5	57.5	CH	3.40	s	57.4	CH	3.49	s	57.5	CH	3.35	s	57.0	CH	3.51	s
6	135.4	C			135.9	C			135.7	C			136.1	C		
6′	21.8	CH_3_	1.29	s	22.0	CH_3_	1.53	s	22.9	CH_3_	1.39	s	25.3	CH_3_	1.48	s
7	131.5	CH	5.09	d (8.2)	131.5	CH	5.13	d (6.6)	130.6	CH	5.00	d (9.2)	131.1	CH	5.14	d (7.1)
8	32.9	CH_2_	2.27	m	34.2	CH_2_	2.28	m	34.1	CH_2_	2.27	m	33.8	CH_2_	2.27	m
9	36.3	CH_2_	2.29	m	35.5	CH_2_	2.32	m	40.4	CH_2_	2.23	m	40.6	CH_2_	2.38	m
10	132.1	C			131.1	C			131.6	C			131.4	C		
10′	22.0	CH_3_	1.31	s	21.8	CH_3_	1.33	s	22.9	CH_3_	1.31	s	21.7	CH_3_	1.35	s
11	123.2	CH	6.17	s	123.1	CH	6.08	s	122.9	CH	6.11	s	122.8	CH	6.15	s
12	46.4	C			46.2	C			42.0	C	2.07	br	46.2	C		
12′	22.8	CH_3_	1.52	s	23.0	CH_3_	1.50	s					22.9	CH_3_	1.50	s
13	145.7	CH	6.92	s	146.6	CH	6.92	s	138.6	CH	6.67	s	146.8	CH	6.92	s
14	132.1	C			126.0	C			135.1	C			125.7	C		
14′	168.1	C			167.7	C			167.5	C			168.5	C		
15	27.8	CH	2.97	m	28.0	CH	2.93	m	27.8	CH	2.95	m	28.0	CH	2.91	m
15′	13.1	CH_3_	1.33	m	13.4	CH_3_	1.34	m	13.8	CH_3_	1.30	m	13.2	CH_3_	1.32	m
16	32.9	CH_2_	1.79	br	33.23	CH_2_	1.74	br	27.8	CH_2_	1.58	br	33.1	CH_2_	1.69	br
			2.71	dd (14.8, 7.9)			2.65	dd (14.4, 7.9)			2.23	m			2.65	dd (14.6, 7.9)
17	85.9	C			85.6	C			82.7	C			85.6	C		
18	198.6	C			199.3	C			202.1	C			200.3	C		
19	55.7	CH	3.82	br	55.5	CH	3.67	br	42.1	CH	3.71	br	42.1	CH	3.60	br
20	124.8	CH	4.90	s	125.6	CH	4.93	s	141.5	C			125.8	CH	4.92	s
20′									14.3	CH_3_	1.48	s				
21	140.9	C			140.2	C			120.3	CH	4.95	d (2.3)	140.4	C		
21′	19.2	CH_3_	1.69	s	19.2	CH_3_	1.69	s					18.9	CH_3_	1.69	s
22	39.59	CH_2_	2.29	m	39.9	CH_2_	2.20	m	34.1	CH_2_	2.32	m	39.7	CH_2_	2.26	m
			1.94	m			1.97	m			1.19	m			1.95	m
23	84.4	CH	3.33	dt (10.4, 4.8)	85.0	CH	3.36	dt (10.2, 4.8)	84.56	CH	3.41	dt (10.4, 4.8)	84.9	CH	3.39	dt (10.5, 4.8)
24	46.2	CH	1.94	m	46.7	CH	1.90	m	46.8	CH	1.93	m	46.5	CH	1.90	m
25	35.3	CH_2_	1.34	br	35.6	CH_2_	1.31	br	36.4	CH_2_	1.52	br	37.8	CH_2_	1.54	br
26	42.6	CH	2.08	br	43.1	CH	2.09	br	37.9	CH	2.07	br	42.9	CH	2.09	br
26′	22.8	CH_3_	0.68	d (5.6)	23.1	CH_3_	0.70	d (5.1)	22.7	CH_3_	0.68	d (5.9)	22.9	CH_3_	0.69	d (5.7)
27	103.6	CH	4.67	d (7.9)	103.4	CH	4.77	d (7.9)	103.1	CH	4.81	d (7.9)	103.2	CH	4.81	d (7.9)
28	70.6	CH	3.79	m	72.4	CH	3.54	dd (7.9, 2.9)	71.9	CH	3.55	dd (7.9, 3.1)	72.0	CH	3.54	dd (7.9, 3.1)
29	73.8	C			70.5	CH	4.33	br	69.6	CH	4.38	br	69.7	CH	4.39	br
29′	19.01	CH_3_	1.22	s												
30	58.4	CH	3.90	d (10.0)	76.1	CH	4.68	dd (9.8, 2.4)	76.8	CH	4.75	dd (9.8, 2.8)	76.8	CH	4.76	dd (9.8, 2.7)
31	75.8	CH	3.33	m	67.6	CH	4.03	m	67.3	CH	4.17	m	67.2	CH	4.18	m
31′	23.7	CH_3_	1.32	d (7.3)	18.2	CH_3_	1.31	d (7.21)	18.4	CH_3_	1.24	d (6.2)	18.3	CH_3_	1.25	d (6.2)
32	170.6	C			164.8	C			171.1	C			170.4	C		
33	113.1	C			108.0	C			106.2	C			106.2	C		
34	139.1	C			144.0	C			144.5	C			144.6	C		
34′	22.6	CH_3_	2.5	s	24.6	CH_3_	2.56	s	24.5	CH_3_	2.54	s	24.3	CH_3_	2.55	s
35	110.0	CH	6.33	s	111.8	CH	6.36	br d	111.7	CH	6.37	d (2.3)	111.7	CH	6.37	d (2.5)
36	161.9	C			162.6	C			165.1	C			165.8	C		
36′					55.9	CH_3_	3.83	s								
37	99.9	CH	6.33	s	97.0	CH	6.45	br d	99.5	CH	6.34	d (2.3)	99.6	CH	6.34	d (2.5)
38	162.9	C			158.7	C			166.0	C			165.3	C		
38′	55.5	CH_3_	3.78	s	55.9	CH_3_	3.82	s	55.8	CH_3_	3.83	s	55.7	CH_3_	3.83	s

**Table 2:** Antiparasitic and cytotoxic activities of tetromycins **1**–**4** and tetromycin B (**5**) (IC_50_, μM).

Compound	*L. major*	*T. brucei brucei* (48 h)	*T. brucei brucei* (72 h)	293T Kidney cells	J774.1 Macrophages
1	>100	29.30	31.69	>100	>100
2	>100	45.39	80.27	>100	50.21
3	36.80	26.90	30.35	33.38	25.72
4	>100	35.85	41.61	58.58	27.54
5	>100	30.87	34.22	71.77	20.20

**Table 3:** Inhibition of various cysteine proteases by tetromycins **3**–**4** and tetromycin B (**5**).

Compound	RD *K*_i_ (μM); *k*_2nd_ (M^−1^ min^−1^)	FP *K*_i_ (μM); *k*_2nd_ (M^−1^ min^−1^)	CL *K*_i_ (μM); *k*_2nd_ (M^−1^ min^−1^)	CB *K*_i_ (μM)	SARS-CoV-PL^pro^ *K*_i_ (μM)
**3**	2.1 ± 0.90; 36,600 ± 1590	1.65 ± 0.25; 617 ± 9	15.0 ± 1.95; 14,977 ± 2005	0.57 ± 0.04	n.d.
**4**	4.00 ± 0.30; 8354 ± 1562	3.10 ± 0.20; 1836 ± 108	22.40 ± 0.80; 14,700 ± 1000	1.60 ± 0.10	40.00 ± 6.50
**5**	0.62 ± 0.03; 14,539 ± 1949	1.42 ± 0.01; 15,540 ± 1295	32.50 ± 0.05; 1576 ± 413	1.59 ± 0.09	69.60 ± 7.20

RD: rhodesain; FP: falcipain-2; CL: cathepsin L; CB: cathepsin B; n.d. not determined; Data presented are average values from at least two independent assays.
